# Questionable Species Names for Distinct Species Clusters: An Empirical Test of the BOLD Molecular Identification Engine

**DOI:** 10.3390/insects16111172

**Published:** 2025-11-17

**Authors:** Elisaveta V. Yakimenko, Anna E. Romanovich, Vladimir A. Lukhtanov

**Affiliations:** 1 Department of Karyosystematics, Zoological Institute, Russian Academy of Sciences, Universitetskaya Nab. 1, 199034 St. Petersburg, Russia; 2 Department of Entomology, St. Petersburg State University, Universitetskaya Nab., 7/9, 199034 St. Petersburg, Russia; 3 Resource Center for Development of Molecular and Cellular Technologies, St. Petersburg State University, Universitetskaya Nab. 7/9, 199034 St. Petersburg, Russia; aromanovich@gmail.com

**Keywords:** *COI*, DNA barcoding, *Lepidoptera*, *Papilionoidea*, species identification, taxonomy

## Abstract

DNA barcoding is an effective method for species identification, but its practical application, as implemented in the Barcode of Life Data System (BOLD), faces numerous challenges. In our work, we conducted an empirical test of this approach using butterflies of the Volga River region in eastern Europe as a model system. We demonstrate that DNA barcoding is a powerful tool for identifying species clusters of the local fauna studied. However, assigning the identified clusters to scientific species names using BOLD was problematic for more than half of the species analyzed. The reasons for these problems are numerous errors in (1) species and even (2) generic identifications of DNA barcodes in the BOLD database (30% and 26% of all problematic cases, respectively), (3) similarity of DNA barcodes in different species (22%), (4) unresolved taxonomic problems associated with the species names that BOLD suggests as identifications (18%), (5) anomalous barcodes (2%), and (6) incompleteness of the BOLD database (2%). Solving problems 1, 2 and 5 requires improving the DNA barcode curation system and minimization of the identification errors in the BOLD database. Problems 3 and 6 can be partly solved by accumulating DNA barcodes, especially barcodes of local faunas, since populations of different species with identical DNA barcodes often have non-overlapping areas. Problem 4 is the most difficult and requires further intensive taxonomic research to solve it.

## Simple Summary

DNA barcoding is a powerful methodology and a tool in the field of species identification and discovery, significantly contributing to progress in various fields of biology, including evolutionary studies, integrative taxonomy, and even genomics. Despite this, there are many problems in both the theory and practical application of DNA barcoding in biology. In our study, we conduct an empirical test of the performance of a DNA barcoding-based identification system in terms of discovering species clusters and issuing scientific Linnaean binomial names. To our surprise, we found an extremely high rate of incorrect or questionable identifications. We analyze the causes of these errors and suggest recommendations on how the molecular species identification system can be improved.

## 1. Introduction

DNA barcoding is a biological technique that enables species identification of organisms based on comparison of short DNA fragments [1]. Over the past 20 years, DNA barcoding research has become one of the most popular areas of biology and biotechnology. Hundreds, if not thousands, of papers are published annually, devoted to both general issues of DNA barcode application and descriptions of DNA barcoding successes for species identification of various groups of organisms in different regions of the Earth (e.g., [2,3,4,5,6]). Many of these papers follow approximately the same pattern and describe, using a wide variety of organisms as examples, a large difference between intra-specific and inter-specific variations, resulting in a significant barcoding gap between species and leading to successful species identification [1,2,7,8,9]. Overall, the enormous success of DNA barcoding is beyond doubt, and one of the monumental proofs of this is the Barcode of Life Data System (BOLD) (https://boldsystems.org/) (accessed on 6 November 2025). BOLD is a giant database and a real working machine that, receiving DNA barcodes as input, produces species identifications as output. BOLD does this directly in the form of binomial Linnaean names, as well as in the form of identification trees on which the sample in question is located, surrounded by the closest sequences [10].

Species identification, both in traditional taxonomy and in BOLD work, involves two procedures. The first is identifying clusters of similar individuals that are presumably conspecific, i.e., belong to the same species. The second is finding and assigning a valid scientific name to the identified clusters. A specimen that is not assigned to a scientific species name, or that is assigned to an incorrect species name, is of limited value in subsequent research and practical applications or, worse, can be a source of subsequent errors. BOLD has the ability to complete both procedures. First, a specimen can be assigned to a specific BIN (the Barcode Index Number), a cluster of similar sequences. The BINs can be interpreted as operational taxonomic units that roughly correspond to species. Second, the specimen can be assigned to a taxonomic species and its valid scientific name [11]. In BOLD, the first procedure is based on strict algorithms [10,11]. The second procedure is more subjective, since obtaining a scientific name is based on those species identifications that are already contained in the BOLD database and were made earlier by submitters with different taxonomic qualifications (and sometimes without them). Even experts in taxonomic groups can make mistakes in species identification. The rate of such errors increases when non-experts and amateurs submit sequences to BOLD, providing them with their identifications.

Studies devoted to DNA barcoding usually focus on the first procedure. These studies have generally confirmed the presence of a barcoding gap between the vast majority of species in the analyzed regions and taxonomic groups (e.g., [1,2,7,8,9,12]), although some groups of organisms may represent exceptions [13]. There are significantly fewer studies that address the problems of the second procedure and analyze the correctness of the resulting species identifications (see, for example, [14,15]).

In our work we focus on the analysis of the second DNA barcoding procedure, studying the local fauna of butterflies (Lepidoptera, Papilionoidea) of the Ulyanovsk region that geographically belongs to the Middle Volga territory, located in the east of Europe. According to the regionalization accepted by many biologists and geographers (see [16]), the Ulyanovsk region is part of the so-called Volga-Ural region, occupying vast areas in the extreme south-east of the European continent. In terms of the butterfly DNA barcodes, the Volga-Ural region has been studied only fragmentarily. The BOLD (https://boldsystems.org/) (accessed on 6 November 2025) and NCBI (https://www.ncbi.nlm.nih.gov/genbank/) (accessed on 27 August 2025) databases contain DNA barcodes of some Volga-Ural species that were obtained accidentally either during larger projects [17,18] or during taxonomic and biogeographical studies of individual genera [19,20]. This contrasts with the better-studied neighboring regions of Western Europe [21,22], Kazakhstan and Central Asia [23], and with more distant territories such as the USA and Canada [24].

The analyzed region lacks mountains, and its lepidopteran fauna is represented almost exclusively by widespread Trans- and Western Palearctic species. Therefore, despite the region’s poor study, given the existence of extensive barcode libraries for neighboring areas, it could be expected that all collected specimens would be quickly and reliably identified based on their barcodes. This expectation was largely met in the sense that, using the BOLD identification mechanism, we only encountered one situation where we could not find identical or similar DNA barcodes to compare with the sample under study. However, to our surprise, when attempting to obtain Linnaean scientific names for the collected specimens, we encountered problems for more than half of the species analyzed. The reasons for these problems were numerous errors in (1) species and even (2) generic identifications of DNA barcodes in the BOLD database (30% and 26% of all problematic cases, respectively), (3) similarity of DNA barcodes in different species (22%), (4) unresolved taxonomic problems associated with the species names that BOLD suggests as identifications (18%), (5) anomalous barcodes (2%), and (6) incompleteness of the BOLD database (2%). We argue that the main way to solve these problems is to accumulate information on DNA barcodes of local faunas and minimize species identification errors in the BOLD database.

## 2. Materials and Methods

The material was collected by the authors and by students of Ulyanovsk State Pedagogical University in the period from 2020 to 2024 in different localities of Ulyanovsk region and adjacent areas of Saratov region (Supplementary Materials, Table S1). The samples selected for the study were not subjected to any chemical treatment before drying. DNA was isolated from one or two butterfly legs or from a piece of the abdomen using the CTAB DNA extraction protocol [25]. To amplify a 658 bp fragment of the mitochondrial *cytochrome c oxidase subunit I* gene (*COI*), primers LEP-F1, 5′-ATTCAACCAATCATAAAGATAT-3′; and LEP-R1, 5′-TAAACTTTCTGGATG TCCAAAAA-3′ [7] were used. Amplification was performed by polymerase chain reaction (PCR). The volume of the mixture was 15 μL and contained: 9.9 μL ddH20; 0.3 μL of each primer pair (LepF/LepR); 3 μL ScreenMix (Eurogen, Moscow, Russia) and 1.5 μL of matrix DNA. PCR was performed on a SimpliAmp DNA amplifier (ThermoFisher Scientific, Waltham, Massachusetts, USA) in automated mode. Initial denaturation was 3 min at 94 °C, followed by 35 by cycles of denaturation, annealing, and extension. Denaturation temperature was 94 °C for 30 s. Annealing temperature was 50 °C for 30 s. Extension temperature was 72 °C for 45 s. Final extension step was 10 min at 72 °C. Hold temperature was 4 °C.

PCR products were subjected to Sanger sequencing using the same primers as for amplification. Sequencing of the PCR-products was carried out at the Research Resource Center for Molecular and Cell Technologies (Scientific Park, St. Petersburg State University, St. Petersburg, Russia) using ABI 3500xL analyzer (Applied Biosystems, San Francisco, CA, USA). The obtained sequences were uploaded to GenBank (accession numbers PQ611771-PQ611914). To identify clustering of samples, a phylogenetic tree was constructed using the MrBayes v.3.2.7 program [26]. For this purpose, we first determined the optimal nucleotide substitution models for the entire alignment, as well as separately for the first, second, and third codon positions using the MEGA 11 program [27]. The following optimal models were identified: GTR + G for the entire alignment, HKY + G for the first codon position, HKY + G for the second codon position, and GTR + G for the third codon position. All these models correspond to the same settings nst = 6 and rates = gamma in the MrBayes program. Therefore, the entire matrix was analyzed as a single partition with the setting nst = 6 and rates = gamma. Two runs of 10,000,000 generations with four chains (one cold and three heated) were performed. The level of convergence was monitored using average standard deviation of split frequencies parameter (was 0.006 after 10,000,000 generations indicating a high level of convergence). The first 25% trees were discarded prior to computing a consensus phylogeny and posterior probabilities. The consensus of the obtained trees was visualized using FigTree 1.4.4 (http://tree.bio.ed.ac.uk/software/, accessed on 23 October 2025). The samples belonging to hawk moths family (Sphingidae) were taken as an outgroup to root the tree.

Morphological identification of each sample was carried out using the keys given in the monograph A.L. Lvovsky and D.V. Morgun [28], color images in the monograph on the fauna of butterflies of Russia and adjacent territories [29], and then checked using the collection of the Zoological Institute of the Russian Academy of Sciences in St. Petersburg, Russia. Molecular identification of the DNA barcodes was performed in the BOLD system, version 5 (https://id.boldsystems.org/) (accessed on 6 November 2025) using (1) the most similar sequence search algorithm and (2) the identification tree construction algorithm. To search for similar barcodes, the public animal library BOLD database was used. As of 6 November 2025, it included 8,938,256 non-redundant *COI* sequences of at least 500 bp within the 648 bp barcode region. The search was performed using Rapid Species Search Operating Mode with settings that find up to 25 most similar sequences (search depth up to 94% similarity). Identification trees were built in BOLD with default settings (Data Type: Nucleotide; Distance Model: Kimura 2 Parameter; Marker: *COI*-5P; Codon Positions: 1st, 2nd, 3rd).

## 3. Results

**Figure 1:**
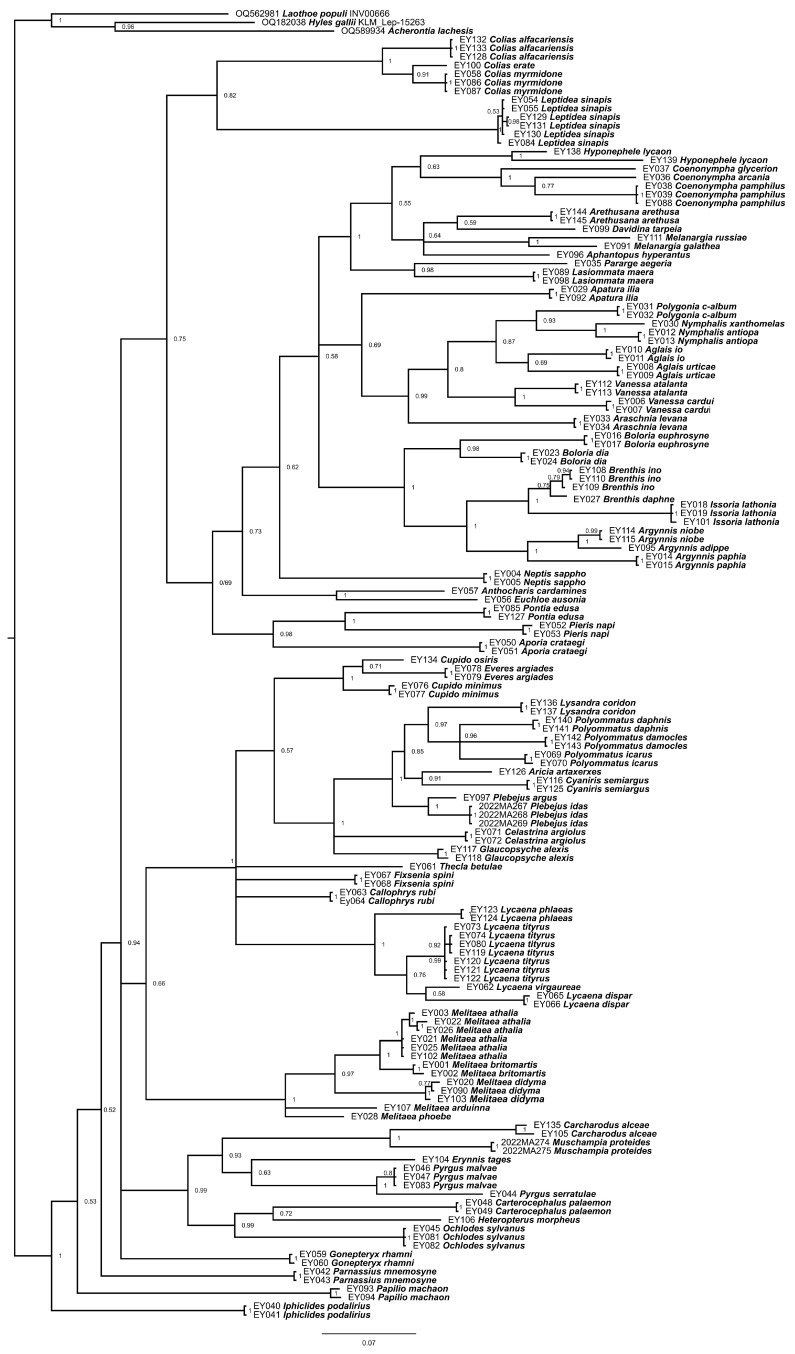
Bayesian phylogenetic tree (50% consensus) of the studied mitochondrial DNA barcodes. Node values indicate Bayesian posterior probabilities. Scale bar = 0.07 substitutions per position. The *COI* barcodes of *Laothoe populi*, *Hyles gallii* and *Acherontia lachesis* belonging to the family Sphingidae were used to root the tree.

We obtained 149 DNA barcodes belonging to 75 butterfly species (Supplementary Materials, Table S1). These barcodes were compared with each other, as well as with all other barcodes of these 75 species and other most closely related samples in the BOLD database. Barcode analysis showed that the studied samples are clustered into 76 discrete groups, each of which is characterized by a high genetic distance to the nearest neighbor (>3%), high (100%) support on the Bayesian tree (if the group included two or more individuals) and a large length of branches connecting each of these 76 units with the nearest neighbor on the Bayesian tree (Figure 1). 74 of the 76 groups fully corresponded to the species identified based on morphological features. For one species (*Hyponephele lycaon* (Rottemburg, 1775)), two studied individuals fell in two different groups, with a distance of 7.2% between them.

In the second stage of DNA identification, an attempt was made to obtain scientific species names for these 76 clusters using the BOLD system. In 46% of cases (35 out of 76 clusters), we obtained one variant of molecular species identification, which completely corresponded to the determination based on morphology. In these error-free identifications, the haplotypes of the mitochondrial *COI* fragment found in butterflies of the Middle Volga were either identical to the haplotypes previously found in these species in other regions, or differed in one or more substitutions, but confidently (BPP = 100) fell into the previously identified species clusters.

In 54% of cases (41 out of 76), identifications were problematic. This was reflected in the fact that DNA barcodes were identified only to the genus (not to the species) or the species identification confidence was less than 100%. Details and reasons for all problematic species identifications are provided in Table 1 and Table S2, and the most characteristic and typical examples of problematic species identifications are described below. It should be emphasized that for all but one species, there were multiple similar barcodes in BOLD for each studied specimen from the Volga region. Thus, in 98.7% of cases, identification problems were not due to an incomplete BOLD database (i.e., a lack of specimens with identical or similar barcodes).

*Erynnis tages* (Linnaeus, 1758) (family Hesperiidae). A haplotype identical to that of nine individuals from Western Europe was found in Ulyanovsk region. In addition, in the BOLD database, this haplotype is also attributed to one specimen (ATLAS092-22) identified as *Colias alfacariensis* Ribbe, 1905 (family Pieridae) (Table S2). Unfortunately, the BOLD database lacks an image of this specimen, making it impossible to verify its identity based on the wing pattern. Clearly, this identification is either the result of contamination or an inaccurate taxon labeling (misprint) in the documentation of this specimen in BOLD.

*Carcharodus alceae* (Esper, 1780) (Hesperiidae). Two haplotypes were found, differing by eight nucleotide substitutions. One haplotype is identical to that of individuals from France and Spain. In addition, in the BOLD database, similar haplotype (similarity of 99.53%) is also attributed to a sample (EULEP779-15-22) identified as *Erynnis tages* (Hesperiidae), i.e., as a representative of a different genus (Table S2). Unfortunately, the BOLD database lacks an image of this specimen, making it impossible to verify its identity based on the wing pattern. Clearly, this identification is either the result of contamination or an inaccurate taxon labeling (misprint) in the documentation of this specimen in BOLD.

For the second haplotype, BOLD returns four identifications with comparable frequency (*C. floccifera* (Zeller, 1847), *C. stauderi* (Reverdin, 1913) *C. alceae*, and *Carcharodus* sp.) (Table S2). These identifications are based on vouchers that have 100% similarity to the second haplotype, which may indicate that the species are incorrectly identified by the authors, or that there are problems with the taxonomy of this group, or both.

*Muschampia proteides* (Wagner, 1929) (Hesperiidae). Two studied individuals were found to have two haplotypes that differed by one nucleotide substitution. According to BOLD, both haplotypes are identical or similar to several species of the genus *Muschampia*: *M. sovietica* Sichel, 1964 from Russia (Volgograd region), *M. proteides* from Turkey and *M. proto* (Ochsenheimer, 1808) from Crimea (Table S2).

*Pyrgus malvae* (Linnaeus, 1758) (Hesperiidae). The studied individuals were found to have a haplotype identical to that of individuals from Western Europe. The same haplotype is also found in *P. melotis* (Duponchel, 1834) from Israel, and a similar haplotype (99.22%) is found in *P. malvoides* (Elwes et Edwards, 1897) from Spain (Table S2). However, the use of this haplotype for identification of butterflies of the Ulyanovsk region seems possible, since the last two species are not found in the Volga-Ural region.

*Iphiclides podalirius* (Linnaeus, 1758) (Papilionidae). Two studied individuals had a haplotype identical to that of *I. feisthamelii* (Duponchel, 1832) specimens from Spain, France, and Portugal, as well as *I. podalirius* from France, Spain, Portugal, Kazakhstan, Russia, Turkey, and Iran (Table S2). The presence of identical haplotypes in these species was previously explained by mitochondrial introgression, during which the mitochondria of *I. podalirius* were transferred to *I. feisthamelii* [30]. The obtained haplotype can be used to identify butterflies from the Ulyanovsk region, since *I. feisthamelii* is endemic to southwestern Europe and is not found in the Middle Volga region.

*Euchloe ausonia* (Hübner, 1805) (Pieridae). The only studied individual had a haplotype identical to that of specimens from Greece and Serbia. According to BOLD, similar haplotypes are found in two more species of the genus *Euchloe*, *E. persica* Verity, 1908 from Iran (99.67% similarity) and *E. pulverata* (Christoph, 1884) (99.69% similarity) from Turkmenistan. In addition, the BOLD database contains individuals identified as *Euchloe ausonia*, which have 98% similarity to this haplotype. These individuals no longer have gaps from the species *E. ausonides* (Lucas, 1852), *E. ochracea* (Trybom, 1877) and *E. pulverata* (Table S2). In this context, the identification of all these species by DNA barcodes is problematic.

*Pontia edusa* (Fabricius, 1777) (Pieridae). Two haplotypes were found differing in two nucleotide substitutions. According to BOLD, both haplotypes can be attributed to both *P. edusa* and *P. daplidice* (Linnaeus, 1758) (Table S2). In fact, these two cryptic species are clearly distinguished by molecular markers, including the *COI* gene [31]. Therefore, the identification “*P. daplidice*” for individuals from the BOLD database, given in Table S2, is most likely erroneous. Both found haplotypes can be used to identify the species in the Ulyanovsk region, taking into account the geographical criterion. True *P. daplidice* is found only in North Africa, the Iberian Peninsula, southern Italy, and the Middle East, while *P. edusa* inhabits the rest of the Palearctic [31].

*Colias erate* (Esper, 1805) (Pieridae). The only studied individual had a haplotype identical or similar to that of *C. erate* from the Czech Republic, Ukraine, Kyrgyzstan, Romania, Austria, Pakistan, Kazakhstan, *C. crocea* (Geoffroy, 1785) from Armenia, Spain, Italy, Romania, and Greece, *C. marnoana* Rogenhofer, 1884 from Sudan, and *C. poliographus* Motschulsky, 1861 from China (Table S2). Species identification of *C. erate* based on DNA barcoding is impossible, since both *C. erate* and *C. crocea* are found in the Ulyanovsk region [16].

*Colias myrmidone* (Esper, 1780) (Pieridae). Three studied individuals had a haplotype identical to that of individuals from Romania, the Czech Republic, and European Russia. The same haplotype was also found in *C. caucasica* Staudinger, 1871 from North Macedonia and Serbia (EULEP2757-15, EULEP3694-16) (Table S2). It should be noted that although males of these two taxa differ in the intensity of red coloration and can be distinguished, the taxonomy of this group is far from clear [32]. We checked the BOLD images of these two species and can confirm that the BOLD identifications for these two taxa are correct. Thus, the identity of the barcodes in this case is not the result of misidentification. Despite this identity, the use of the discovered haplotype for identification of butterflies from the Ulyanovsk region seems possible, since *C. caucasica* is not found in the Volga-Ural region [16].

*Coenonympha arcania* (Fabricius, 1761) (Nymphalidae). A haplotype identical or similar to other *C. arcania* sequences in the BOLD database was found in the Ulyanovsk region (Table S2). This haplotype has a high similarity to two other species of the genus *Coenonympha*, *C. leander* (Esper, 1784) (sample EULEP481-14, 99.68%) and *C. orientalis* Rebel, 1910 (sample EULEP1079-15, 99.36%).

*Hyponephele lycaon* (Nymphalidae). Two haplotypes were found in two studied individuals that differ by 44 nucleotide substitutions. According to BOLD, one haplotype (EY138) is found in *H. lycaon* from Russia and Western Europe, as well as in *H. przhewalskyi* Dubatolov, Sergeev et Zhdanko, 1994 from Kyrgyzstan (Table S2). In addition, in the BOLD database, similar (99.84%) haplotype is attributed to a sample (BGEPL1865-25) identified as *Coenonympha pamphilus* (Linnaeus, 1758) (Nympalidae), i.e., as a representative of a different genus. Unfortunately, the BOLD database lacks an image of this specimen, making it impossible to verify its identity based on the wing pattern. Clearly, this identification is either the result of contamination or a typographical error in the documentation of this specimen.

Another haplotype, differing by 7.2%, belongs to specimen EY139, which was collected in exactly the same locality and on the same day as EY138. This haplotype forms a distinct clade together with the following similar, but not identical, sample from Spain (99.53%, BDE426-19) and more distantly related samples from Iran and Israel (98.11–98.26%) (Table S2, Figure S1). Based on such a high level of differentiation and clustering, one might expect the latter samples to belong to a different species. However, analysis of the morphology of these butterflies and their nuclear genes (e.g., https://www.ncbi.nlm.nih.gov/nuccore/MW198386, accessed on 6 November 2025) proves that they do not belong to a separate species, but rather represent a group of haplotypes with abnormally high differentiation.

*Davidina tarpeia* (Pallas, 1771) (Nymphalidae). A haplotype identical to that of *D. tarpeia* individuals from Bashkortostan, Orenburg and Chelyabinsk regions, Altai Krai and Buryatia (Russia) was found. The same haplotype is found in two other species of the genus *Davidina*, *D. lederi* (Alpheraky, 1897) from Mongolia and *D. dzhulukuli* (Korshunov, 1998) from Altai (Russia) (Table S2). However, the use of this haplotype for identification of butterflies of the Ulyanovsk region seems possible, since *D. lederi* and *D. dzhulukuli* are not found in the Volga-Ural region [16].

*Apatura ilia* (Denis et Schiffermüller, 1775) (Nymphalidae). Two haplotypes differing in one nucleotide substitution were found. One haplotype is identical to the haplotypes of specimens from Austria, Finland, Spain, Slovakia, and the Czech Republic. The other haplotype is identical to specimens from Russia (Belgorod Region), Belarus, Ukraine, Spain, Estonia, Serbia, Austria, Romania, and Bulgaria. Both haplotypes also have a high similarity with known *COI* sequences of *A. metis* (Freyer, 1829) (99.53–99.69%) (Table S2). On the identification tree, DNA barcodes of these two species are intermixed, without forming species-specific clusters (Figures S2 and S3). Therefore, distinguishing these two species by DNA barcodes is not possible. Since *A. ilia* and *A. metis* are sympatric and *A. metis* is found in the adjacent areas of the lower Volga [16], identification of species of the genus *Apatura* is problematic in the Ulyanovsk region.

*Neptis sappho* (Pallas, 1771) (Nymphalidae). Two haplotypes differing in two nucleotide substitutions were found. One haplotype is identical to *N. sappho* haplotype from Austria and Ukraine, as well as to one specimen (EZHBA475-07) labeled in BOLD as a species from another genus, *Araschnia levana* (Linnaeus, 1758) (Nymphalidae). Based on photo in BOLD, this sample belongs to *N. sappho*, thus representing a case of a mislabeled specimen or a case of contamination (Table S2).

Another haplotype is not identical to haplotypes in individuals from Russia and other countries, differing from the closest haplotype from Russia (Bashkortostan) by one nucleotide substitution; however, its clustering with other *N. sappho* specimens in the BOLD database allows it to be used for species identification.

*Melitaea arduina* (Esper, 1783) (Nymphalidae). The only studied individual had a haplotype identical to that of individuals from Russia (Chelyabinsk region), Serbia, Romania, Kazakhstan, North Macedonia, and Ukraine. According to BOLD, the same haplotype is found in another species of this genus, *M. cinxia* (Linnaeus, 1758) from Russia (Chelyabinsk region) (sample MBMPA271-09), which is probably due to an error in identification. Unfortunately, BOLD lacks a photograph of this specimen, preventing verification of the identification. Furthermore, the BOLD database contains a barcode from Kyrgyzstan (GWOUO994-24) with a 98.9% similarity and identified as *M. cinxia*, although it is illustrated with a photograph of *Parnassius simonius* (Staudinger, 1889) (Papilionidae) (Table S2). Most likely, this situation is either the result of contamination or an inaccurate labeling in the documentation of the specimen GWOUO994-24.

*Melitaea phoebe* (Denis et Schiffermüller, 1775) (Nymphalidae). The only studied individual had a haplotype similar to haplotypes of other samples from Europe, and also similar to *M. sibina* Alpheraky, 1881 from Kazakhstan (one nucleotide substitution) and *M. ornata* Christoph, 1893 (two nucleotide substitutions) (Table S2). *Melitaea sibina* is known from Central Asia [28] and is not found in the Ulyanovsk region [16]. *Melitaea ornata* is found in the Volga-Ural region sympatric with *M. phoebe* [16]. Therefore, the use of the obtained haplotype for species identification in the region is problematic.

*Argynnis niobe* (Linnaeus, 1758) (Nymphalidae). The studied individuals were found to have a haplotype identical to the haplotype found in *A. niobe* from Spain and Greece, *A. aglaja* (GBCAB4456-24), *A. adippe* (EZHBA094-07, EZHBA096-07) from Russia (Kemerovo region), and *A. xipe* Grum-Grshimailo, 1891 (GBMIN85313-17) (Table S2). We cannot confirm or exclude the fact that the mentioned above *A. aglaja*, *A. adippe* and *A. xipe* samples are identification errors. Unfortunately, the BOLD images of these problematic specimens are missing or incomplete (only the underside of the wings), making it impossible to verify the identification. Generally, molecular identification of *A. niobe* currently seems problematic.

*Callophris rubi* (Lynnaeus, 1758) (Lycaenidae). The studied individuals were found to have a haplotype found in *C. rubi* from different countries of Western Europe, Russia, and Ukraine, as well as in *C. chalybeitincta* Sovinsky, 1905 from Russia (Table S2).

## 4. Discussion

Within the studied samples from the Middle Volga, there were no individuals of different species with identical or similar DNA barcodes, and on the tree, each species forms a clade characterized by high support. This confirms that DNA barcoding is a powerful method for precisely distinguishing species clusters [1,2,7,8,9]. However, at the second stage of DNA barcoding, when trying to obtain scientific species names based on the BOLD database and the BOLD identification engine, it turned out that 40 of the 75 studied species (53%) were problematic. This is significantly more than 5%, which is usually positioned as the standard level of the proportion of species not determined by DNA barcoding [1,2,7,8,10,11]. A higher proportion of species undetectable by mitochondrial DNA barcodes has been previously reported for some organisms with rapid speciation and/or high rates of interspecific hybridization. These include, for example, butterflies of the subgenus *Agrodiaetus* [13], in which rapid speciation occurs due to extremely rapid chromosomal evolution [33]. Another example is African cichlid fishes, in which speciation is driven by a combination of natural and sexual selection leading to rapid diversification in species-specific traits such as diet, body shape, and mating coloration [34,35]. It is important to note that all of these previous studies, which yielded high or low rates of species identification success, were aimed at analyzing genetic discontinuities between species. They did not study the problems of finding valid species names for the detected clusters. Only a few works have attempted to analyze the problems associated with the imperfection of the database and the algorithms for species identification in BOLD [36].

Our analysis shows that, despite the clear clustering of all local faunal samples, matching these clusters with correct species names obtained using BOLD algorithms poses significant challenges. The proportion of problematic species identifications obtained using the BOLD algorithm was significantly higher than expected. Below, we classify these problematic cases into the following six groups.

### 4.1. Species Identification Errors in the BOLD Database (Problems from a Same Genus)

The most common problem we encountered was errors in species identifications of DNA barcodes in the BOLD database. BOLD is rife with such errors, so addressing them is an obvious priority. In most cases, misidentified specimens are sporadically found among correct identifications. However, for example, in the case of *Carcharodus alceae*, the frequency of probable erroneous (incorrect species indicated) or inaccurate (species not indicated at all) identifications was high and comparable to the frequency of probable correct identifications.

Identifying candidates for such errors is a relatively straightforward procedure, since the mere presence of two or more species identifications for a single barcode group already indicates a potential error. However, the problem is complicated by the fact that the same pattern is observed in the case of undifferentiated barcodes and mitochondrial introgression. Elimination of such errors is quite difficult and may require rechecking not only the images of these specimens but also the voucher specimens themselves.

### 4.2. Possible Contamination/Inaccurate Labeling (Errors in Identification of Genera and Families)

We were surprised to discover that the second most common problem with the BOLD engine was DNA barcodes being misidentified at the genus or even family level. Such gross errors most likely occur due to carelessness in taxon labeling or typos when submitting barcodes to BOLD or due to contamination in the laboratory, leading to the amplification of foreign DNA. While analyzing our samples from the Middle Volga region, we found several cases where BOLD, in addition to correct identification, returned errors at the genus and family level. For example, *Erynnis tages* (Hesperiidae) was identified as *Colias alfacariensis* (Pieridae), *Carcharodus alceae* (Hesperiidae) as *Erynnis tages* (Hesperiidae), and *Neptis sappho* (Nympalidae) as *Araschnia levana* (Nympalidae).

In all cases, these erroneous identifications were in the minority. However, such rare errors can be confusing to an inexperienced user. Fortunately, errors of this kind are easily detected and can be easily removed from the BOLD when curating this database.

### 4.3. Undifferentiated DNA Barcodes

According to the literature, undifferentiated DNA barcodes are the most common problem of species identification using DNA barcoding methodology [13]. In our study, this situation was the third most common obstacle to species identification. It is interesting to note that in 5 out of 13 cases, identical or very close DNA barcodes were found in species whose ranges do not even come close to the Middle Volga. For example, this situation applies to the Volga populations of *Pyrgus malvae* (identical or close haplotypes are found in *P. melotis* from Israel and *P. malvoides* from Spain), *Iphiclides podalirius* (identical or close haplotypes are found in *I. feisthamelii* from the Iberian Peninsula), *Colias myrmidone* (identical or close haplotypes are found in *C. caucasica* from the Balkan Peninsula), *Davidina tarpeia* (identical or close haplotypes are found in *D. lederi* from Mongolia and *D. dzhulukuli* from Altai), and *Papilio machaon* (similar haplotypes are found in *P. saharae* from North Africa). Theoretically, identification of such species of local fauna (in this case, the Middle Volga) by DNA barcodes is possible given that similar species inhabit remote regions. However, this does not mean that species of these complexes can be discriminated in all other regions. For example, *Iphiclides podalirius* and *I. feisthamelii* are parapatric in Western Europe [30]. *Davidina tarpeia* occurs together with *D. lederi* and *D. dzhulukuli* in Altai and Mongolia [29]. Thus, identification of these species by barcodes becomes problematic in other regions. This emphasizes the importance of refining the ranges and creating libraries of DNA barcodes of local faunas for species identification. In 4 out of 14 cases, identical or very close DNA barcodes were found in species whose ranges overlap in the Middle Volga (*Colias erate/C. crocea*, *Coenonympha arcania/C. leander*) or overlap is probable (*Callophris rubi/C. chalybeitincta*, *A. ilia/A. metis*). Obviously, identification of these species by barcodes in the Middle Volga is impossible.

### 4.4. Existing Taxonomy Uncertainty

There is a situation when DNA barcodes are identical in two or more seemingly different species [37], but the study of these “species” shows that they are not true taxa but conspecific populations of the same species. In this regard, the analysis of *Muschampia proteides* is indicative, for which BOLD gives *M. proto*, *M. sovietica* and *M. proteides* as potential identifications. In fact, as the revision of this complex showed, *M. sovietica* is a synonym of *M. proteides*, and all Eastern European populations previously attributed to *M. proto* are also *M. proteides* [38]. Thus, in this case, the diversity of possible identifications is a consequence of imperfect taxonomy. In fact, these taxonomic problems in the genus *Muschampia* have already been solved [38] but have not yet been taken into account in BOLD.

A similar problem seems to exist with the nominal taxon named *Hyponephele przhevalskyi*, which BOLD presents as one of the possible identifications for one specimen of *H. lycaon*. Although *H. przhevalskyi* was described as a separate species [39], in a later revision it is presented as a taxon conspecific with *H. lycaon* [40].

Less obvious is the situation with the pair of taxa *Colias myrmidone/C. caucasica*, which share common barcodes. We have assigned this pair to category 3 (see above), since these taxa differ morphologically and occupy different ecological niches. However, recent studies raise the question of their possible conspecificity [32].

### 4.5. Anomalous DNA Barcodes

Individuals with abnormally high intraspecific differentiation of DNA barcodes, exceeding intergeneric differentiation, have been described for various organisms, for example, for the butterflies *Colias palaeno* (Linnaeus, 1761) [41] and *Melitaea didyma* (Esper, 1778) [42]. In this study, the samples with abnormal barcodes were shown to exist within the species *Hyponephele lycaon*. Here we do not analyze the causes and mechanisms of occurrence of such abnormal mitochondrial haplotypes, as this is not the purpose of the article. It should only be briefly noted that they are often associated with the *Wolbachia* infection [43,44]. The abnormal *COI* haplotypes create a problem for DNA identification, since these sequences do not cluster together with the DNA barcodes of their conspecifics, creating the illusion of the existence of unidentified or even undescribed cryptic species. From the standpoint of DNA barcoding, it is important to accumulate information on correctly identified anomalous DNA barcodes, which will allow their further use in species identification.

Note: A problem similar in appearance to abnormal barcodes, although fundamentally different in mechanisms, is the presence of NUMTs (nuclear copies of mitochondrial sequences) that can also create problems with DNA identification [45].

### 4.6. Incompleteness of the BOLD Database

The absence of barcodes conspecific with a query is the most obvious and expected problem in species identification via the BOLD engine, especially for poorly studied groups of organisms and rare species. In our analysis, we encountered this situation only when identifying one species, *Polyommatus damocles* (Herrich-Schäffer, 1844), endemic to southeastern Europe [46]. This species was found to be most closely related to *P. altivagans* (Forster, 1956) (98.72%) from Armenia. Additionally, our samples from the Volga have relatively low similarity (98.27%) to samples from Crimea, which are also identified as *P. damocles* in the BOLD database. However, in reality, the Crimean population may represent a separate species, *P. krymaeus* (Sheljuzhko, 1928) [46].

## 5. Conclusions

1. Species identification based on DNA barcode comparison, as implemented in the BOLD database (https://boldsystems.org/) (accessed on 6 November 2025), is very effective; however, it needs to be improved. Our study revealed problems with this approach, which partially coincide with the problems of DNA barcoding of the Hemiptera insects [47]. First of all, these problems are associated with errors in the barcode public data as well as with human errors, such as specimen misidentification, sample confusion, and contamination [47]. It is extremely urgent to work on minimizing errors and typos in species identifications in the BOLD database. Gross errors (at the level of genera and families) can be detected and marked by BOLD staff. Correction of identification errors at the species level usually requires access to voucher specimens and can only be performed by experts in specific groups of organisms. In our opinion, such experts should be coordinators and participants of the relevant BOLD projects. The need for manual, semi-automated or automated curation of the DNA barcode databases to improve DNA identifications is well recognized [15,48,49]. However, the curation procedure is not yet integrated into the standard BOLD system algorithm. We recommend creating such an algorithm in BOLD, where, upon detection of conflicting identifications, the system automatically sends a request for clarification to the participants of the corresponding BOLD projects.
2. In many cases, even when individuals of different species share DNA barcodes, individuals of local faunas can be unambiguously identified if a geographic correction is introduced. Such a correction can be based on the fact that an identical DNA barcode of another species is found only in a different geographic region. For this reason, DNA barcoding of not only global and regional but also local faunas and floras should be welcomed. However, it should be kept in mind that this approach should be used with caution, since the most complete species lists may contain gaps and species may be found far beyond their known ranges [50]. Unrecognized cryptic species, as well as migrations and range expansions of species, can create additional problems.
3. One of the problems of molecular species identification is the identity or high similarity of DNA barcodes in different species. However, as our analysis shows, the identity of DNA barcodes in nominally different species may be a consequence of their real conspecificity. In other words, units formally described as different species may belong to the same evolutionary lineage and actually represent a single species. Recognition of such cases requires further intensive research in the field of classical and integrative taxonomy. Although this type of work is far beyond the scope of the pure DNA barcoding studies, DNA barcoding can be an incentive for them. In cases of poorly studied, formally described taxa, the identity of DNA barcodes may indicate the need for their further analysis and testing for conspecificity.
4. Although the incompleteness of DNA barcode libraries may be a major limitation for molecular identification in poorly studied groups of organisms, for well-studied groups such as European butterflies, this problem is of least significance. It may arise when analyzing rare species.

## Figures and Tables

**Table 1:** Problematic identifications (IDs) obtained by BOLD for the studied butterflies of the Volga Region (see Table S2 for the detail).

Query and Its ID Based on Morphology	BOLD ID for the Majority of the Most Similar Barcodes	Other BOLD IDs for the Most Similar Barcodes (Similarity with the Query, %)	Final BOLD ID for the Query Based on the 25 Closest Barcodes	Final BOLD ID Confidence, %	Reasons for BOLD ID Uncertainty	Geographic Correction *
*Aglais io* EY010	*Aglais io*	*Vanessa cardui* (100)	*Aglais* (genus only)	83 (for the genus)	possible contamination/inaccurate taxon labeling	N/A
*Apatura ilia* EY029	*Apatura ilia*	*A. metis* (99.53)	*Apatura* (genus only)	100 (for the genus)	undifferentiated barcodes	No
*Apatura ilia* EY092	*Apatura ilia*	*A. metis* (99.69)	*Apatura* (genus only)	100 (for the genus)	undifferentiated barcodes	No
*Aphantopus hyperantus*EY096	*Aphantopus hyperantus*	*A.* sp. (100), *A. bieti* (99.68)	*Aphantopus* (genus only)	100% (for the genus)	undifferentiated barcodes and/or identification problems	N/A
*Araschnia levana* EY033	*Araschnia levana*	*Boloria selene* (100), *Polygonia c-album* (100)	*Araschnia levana*	92	possible contamination/inaccurate taxon labeling	N/A
*Argynnis paphia* EY014	*Argynnis paphia*	*Polyommatus icarus* (100)	*Argynnis paphia*	96	possible contamination/inaccurate taxon labeling	N/A
*Aricia artaxerxes* EY126	*Aricia artaxerxes*	*Plebejus argus* (100%)	*Aricia artaxerxes*	96	possible contamination/inaccurate taxon labeling	N/A
*Brenthis ino* EY108	*Brenthis ino*	*Melitaea athalia* (99.84%), *B. daphne* (99.68%)	*Brenthis ino*	92	possible contamination/inaccurate taxon labeling; identification problems	N/A
*Callophrys rubi* EY063	*Callophrys rubi*	*C. chalybeitincta* (100), *Novosatsuma collosa* (99.84)	*Callophrys* (genus only)	83 (for the genus)	undifferentiated barcodes	No
*Carcharodus alceae* EY105	*Carcharodus alceae*	*Erynnis tages* (99.53)	*Carcharodus alceae*	96	possible contamination/inaccurate taxon labeling	N/A
*Carcharodus alceae* EY135	*Carcharodus alceae*	*C. floccifera* (100), *C. stauderi* (100), *C.* sp. (100)	*Carcharodus* (genus only)	100 (for the genus)	identification problems	N/A
*Carterocephalus palaemon* EY048	*Carterocephalus palaemon*	*C. silvicola* (99.52)	*Carterocephalus palaemon*	92	identification problems	N/A
*Coenonympha arcania* EY036	*Coenonympha arcania*	*C. leander* (99.68), *C. orientalis* (99.36)	*Coenonympha arcania*	96	undifferentiated barcodes	No
*Coenonympha pamphilus* EY038	*Coenonympha pamphilus*	*C. lyllus* (100)	*Coenonympha pamphilus*	92	existing taxonomy uncertainty	N/A
*Colias erate* EY100	*Colias crocea, C. erate*	*C. crocea* (100), *C. marnoana* (100), *C. poliographus* (99.84)	*Colias* (genus only)	100 (for the genus)	undifferentiated barcodes	No
*Colias myrmidone* EY058	*Colias myrmidone*	*C. caucasica* (100)	*Colias* (genus only)	100 (for the genus)	undifferentiated barcodes	Yes
*Cupido minimus* EY076	*Cupido minimus*	*C. tusovi* (99.84), *C. osiris* (99.84), *Glaucopsyche lycormas* (99.69)	*Cupido* (genus only)	100% (for the genus)	identification problems; existing taxonomy uncertainty	N/A
*Cupido osiris* EY134	*Cupido osiris*	*C. staudingeri* (98.87)	*Cupido* (genus only)	100 (for the genus)	undifferentiated barcodes	N/A
*Cyaniris semiargus* EY116	*Cyaniris semiargus*	*Glaucopsyche lycormas* (100)	*Cyaniris semiargus*	92	identification problems or possible contamination/inaccurate taxon labeling	N/A
*Davidina tarpeia* EY099	*Davidina tarpeia*	*D. dzhulukuli* (100), *D. lederi* (99.66)	*Davidina* (genus only)	100 (for the genus)	undifferentiated barcodes	Yes
*Erynnis tages* EY104	*Erynnis tages*	*Colias alfacariensis* (100)	*Erynnis tages*	96	possible contamination/inaccurate taxon labeling	N/A
*Euchloe ausonia* EY056	*Euchloe ausonia*	*E. pulverata* (99.69), *E. persica* (99.67), *E. ochracea* (98.44)	*Euchloe* (genus only)	100 (for the genus)	existing taxonomy uncertainty and/or identification problems	N/A
*Fabriciana niobe* EY115	*Fabriciana niobe*	*F. adippe* (100), *F. xipe* (100)	*Fabriciana* (genus only)	100 (for the genus)	identification problems	N/A
*Hyponephele lycaon* EY138	*Hyponephele lycaon*	*H. przhewalskyi* (100), *Coenonympha pamphilus* (99.84)	*Hyponephele lycaon*	92	existing taxonomy uncertainty; possible contamination/inaccurate taxon labeling	N/A
*Hyponephele lycaon* EY139	*Different taxa*	different taxa with a low level of similarity	Satyrinae (subfamily only)	100 (for the subfamily)	anomalous barcode	N/A
*Iphiclides podalirius* EY040	*Iphiclides podalirius*	*I. feistahameli* (100)	*Iphiclides* (genus only)	100 (for the genus)	undifferentiated barcodes	Yes
*Leptidea sinapis* EY054	*Leptidea sinapis*	*L. descimoni* (99.84)	*Leptidea sinapis*	92	identification problems	N/A
*Lysandra coridon* EY136	*Lysandra coridon*	*Hipparchia semele* (100)	*Lysandra coridon*	96	possible contamination/inaccurate taxon labeling	N/A
*Melitaea arduinna* EY107	*Melitaea arduinna*	*M. cinxia* (100)	*Melitaea arduinna*	91	identification problems	N/A
*Melitaea britomartis*EU002	*Melitaea britomartis*	*M.* sp. (100), *M. aurelia* (99.84)	*Melitaea* (genus only)	100 (for the genus)	identification problems	N/A
*Melitaea phoebe* EY028	*Melitaea phoebe*	*M. sibina* (99.84), *M. ornata* (99.69)	*Melitaea* (genus only)	100 (for the genus)	undifferentiated barcodes	No
*Muschampia proteides* MA274	*Muschampia proteides*	*M. proto* (100), *M. sovietica* (100), *M.* sp. (100)	*Muschampia* (genus only)	100 (for the genus)	existing taxonomy uncertainty	N/A
*Muschampia proteides* MA275	*Muschampia proteides*	*M. proto* (100), *M. sovietica* (100), *M.* sp. (100)	*Muschampia* (genus only)	100 (for the genus)	existing taxonomy uncertainty	N/A
*Neptis sappho* EY004	*Neptis sappho*	*Araschnia levana* (100)	*Neptis sappho*	94	possible contamination/inaccurate taxon labeling	N/A
*Ochlodes sylvanus* EY045	*Ochlodes sylvanus*	*O. hyrcana* (100), *O. faunus*	*Ochlodes* (genus only)	100 (for the genus)	existing taxonomy uncertainty	N/A
*Papilio machaon* EY093	*Papilio machaon*	*P. saharae* (99.52)	*Papilio machaon*	92	undifferentiated barcodes	Yes
*Pararge aegeria* EY035	*Pararge aegeria*	*Argynnis paphia* (100)	*Pararge aegeria*	95	possible contamination/inaccurate taxon labeling	N/A
*Pieris napi* EY052	*Pieris napi*	*P. rapae* (100)	*Pieris napi*	92	identification problems	N/A
*Plebejus idas* 2022MA269s	*Plebejus idas*	*P. argus* (100), *P.* sp. (100), *P. bellieri* (99.84)	*Plebejus* (genus only)	100 (for the genus)	identification problems	N/A
*Polygonia c-album* EY031	*Polygonia c-album*	*Polygonia interposita* (100), *Aglais io* (100)	*Polygonia* (genus only)	80 (for the genus)	existing taxonomy uncertainty; possible contamination/inaccurate taxon labeling	N/A
*Polyommatus damocles* EY142	different taxa	different taxa with a low (<99%) level of similarity	*Polyommatus* (genus only)	100 (for the genus)	incompleteness of the BOLD database	N/A
*Polyommatus icarus* EY069	*Polyommatus icarus*	*P. juno* (99.69)	*Polyommatus icarus*	96	existing taxonomy uncertainty	Yes
*Pontia edusa* EY085	*Pontia edusa*	*P. daplidice* (100)	*Pontia* (genus only)	100 (for the genus)	identification problems	N/A
*Pontia edusa* EY127	*Pontia edusa*	*P. daplidice* (100)	*Pontia* (genus only)	100 (for the genus)	identification problems	N/A
*Pyrgus malvae* EY046	*Pyrgus malvae*	*P.* sp. (100), *P. malvoides* (99.22)	*Pyrgus* (genus only)	100 (for the genus)	undifferentiated barcodes	Yes

* Yes—In the case of undifferentiated barcodes, species identification in the Volga region is possible given geographic correction (the presence of a closely related taxon in the Volga region is extremely unlikely). No—In the case of undifferentiated barcodes, species identification in the Volga region is impossible given geographic correction (the presence of a closely related taxon in the Volga region is proven or highly probable). N/A—Geographic correction is not applicable, as the problem is not related to undifferentiated barcodes.

## Data Availability

All data supporting the findings of this study are presented in the article and the Supplementary Materials.

## Supplementary Materials

The following supporting information can be downloaded at: https://www.mdpi.com/article/10.3390/insects16111172/s1, Table S1: List of the studied material. Table S2: Results of the BOLD identification for the studied butterflies of the Volga Region. Figure S1: BOLD identification tree for the sample EY139 *Hyponephele lycaon*. Figure S2: BOLD identification tree for the sample EY029 *Apatura ilia*. Figure S3: BOLD identification tree for the sample EY092 *Apatura ilia*.

## Abbreviations

The following abbreviations are used in this manuscript:

BPP	Bayesian posterior probability
ID	Identification

## References

[1] Hebert P.D.N., Cywinska A., Ball S.L., deWaard J.R. (2003). Biological identifications through DNA barcodes. Proc. Biol. Sci..

[2] Hebert P.D.N., Stoeckle M.Y., Zemlak T.S., Francis C.M. (2004). Identification of birds through DNA barcodes. PLOS Biol..

[3] Li X., Yang Y., Henry R.J., Rossetto M., Wang Y., Chen S. (2015). Plant DNA barcoding: From gene to genome. Biol. Rev. Camb. Philos. Soc..

[4] Xu J. (2016). Fungal DNA barcoding. Genome.

[5] Huemer P., Berggren K., Aarvik L., Rennwald E., Hausmann A., Segerer A., Staffoni G., Aspaas A.M., Trichas A., Hebert P.D.N. (2025). Extensive DNA barcoding of Lepidoptera of Crete (Greece) reveals significant taxonomic and faunistic gaps and supports the first comprehensive checklist of the island’s fauna. Insects.

[6] Twyford A.D., Beasley J., Barnes I., Allen H., Azzopardi F., Bell D., Blaxter M.L., Broad G., Campos-Dominguez L., Choonea D. (2024). A DNA barcoding framework for taxonomic verification in the Darwin Tree of Life Project. Wellcome Open Res..

[7] Hebert P.D.N., Penton E.H., Burns J.M., Janzen D.H., Hallwachs W. (2004). Ten species in one: DNA barcoding reveals cryptic species in the neotropical skipper butterfly *Astraptes fulgerator*. Proc. Nat. Acad. Sci. USA.

[8] Barrett R.D.H., Hebert P.D.N. (2005). Identifying spiders through DNA barcodes. Can. J. Zool..

[9] Phillips J.D., Griswold C.K., Young R.G., Hubert N., Hanner R.H. (2024). A Measure of the DNA barcode gap for applied and basic research. Methods Mol. Biol..

[10] Ratnasingham S., Hebert P.D.N. (2007). BOLD: The barcode of life data system. Mol. Ecol. Notes.

[11] Ratnasingham S., Hebert P.D.N. (2013). A DNA-based registry for all animal species: The barcode index number (BIN) system. PLoS ONE.

[12] Raupach M.J., Hannig K., Morinière J., Hendrich L.A. (2020). DNA barcode library for ground beetles of Germany: The genus *Pterostichus* Bonelli, 1810 and allied taxa (Insecta, Coleoptera, Carabidae). ZooKeys.

[13] Wiemers M., Fiedler K. (2007). Does the DNA barcoding gap exist?—A case study in blue butterflies (Lepidoptera: Lycaenidae). Front. Zool..

[14] Janko Š., Rok Š., Blaž K., Danilo B., Andrej G., Denis K., Klemen Č., Matjaž G. (2024). DNA barcoding insufficiently identifies European wild bees (Hymenoptera, Anthophila) due to undefined species diversity, genus-specific barcoding gaps and database errors. Mol Ecol Resour..

[15] Krivosheeva V., Salnitska M., Semerikova D., Gebremeskel A., Ivanova A., Solodovnikov A. (2025). Identification of West Siberian *Quedius* (Coleoptera, Staphylinidae) by *COI* barcodes calls for integrative taxonomy and curation of public DNA libraries. Deut. Entomol. Zeit..

[16] Anikin V.V., Sachkov S.A., Zolotuhin V.V. (2017). Fauna Lepidopterologica Volgo–Uralensis: From P. Pallas to Present Days. Proceedings of the Museum Witt Munich.

[17] Dinca V., Zakharov E.V., Hebert P.D., Vila R. (2011). Complete DNA barcode reference library for a country’s butterfly fauna reveals high performance for temperate Europe. Proc. Biol. Sci..

[18] Dincă V., Dapporto L., Somervuo P., Voda R., Cuvelier S., Gascoigne-Pees M., Huemer P., Mutanen M., Hebert P., Vila R. (2021). High resolution DNA barcode library for European butterflies reveals continental patterns of mitochondrial genetic diversity. Commun. Biol..

[19] Bartonova A., Konvicka M., Korb S., Kramp K., Schmitt T., Fric Z. (2018). Range dynamics of Palaearctic steppe species under glacial cycles: The phylogeography of *Proterebia afra* (Lepidoptera: Nymphalidae: Satyrinae). Biol. J. Linn. Soc..

[20] Krupitsky A.V., Shapoval N.A., Schepetov D.M., Ekimova I.A., Lukhtanov V.A. (2022). Phylogeny, species delimitation and biogeography of the endemic Palaearctic tribe Tomarini (Lepidoptera, Lycaenidae). Zool. J. Linn. Soc..

[21] Dincă V., Montagud S., Talavera G., Hernández-Roldán J., Munguira M.L., García-Barros E., Hebert P.D., Vila R. (2015). DNA barcode reference library for Iberian butterflies enables a continental-scale preview of potential cryptic diversity. Sci. Rep..

[22] Dapporto L., Menchetti M., Voda R., Corbella C., Cuvelier S., Djemadi I., Gascoigne-Pees M., Hinojosa J., Lam N.T., Serracanta M. (2022). The atlas of mitochondrial genetic diversity for Western Palaearctic butterflies. Glob. Ecol. Biogeogr..

[23] Lukhtanov V.A., Sourakov A., Zakharov E.V., Hebert P.D. (2009). DNA barcoding Central Asian butterflies: Increasing geographical dimension does not significantly reduce the success of species identification. Mol. Ecol. Resour..

[24] D’Ercole J., Dapporto L., Opler P., Schmidt C.B., Ho C., Menchetti M., Zakharov E.V., Burns J.M., Hebert P.D.N. (2024). A genetic atlas for the butterflies of continental Canada and United States. PLoS ONE.

[25] Doyle J.J., Doyle J.L. (1987). A rapid DNA isolation procedure for small quantities of fresh leaf tissue. Phytochem. Bull..

[26] Ronquist F., Teslenko M., van der Mark P., Ayres D.L., Darling A., Höhna S., Larget B., Liu L., Suchard M.A., Huelsenbeck J.P. (2012). MrBayes 3.2: Efficient Bayesian phylogenetic inference and model choice across a large model space. Syst. Biol..

[27] Tamura K., Stecher G., Kumar S. (2021). MEGA 11: Molecular Evolutionary Genetics Analysis Version 11. Mol. Biol. Evol..

[28] Lvovsky A.L., Morgun D.V. (2007). Bulavousye Cheshuekrylye Vostochnoy Evropy [Butterflies of Eastern Europe].

[29] Tuzov V.K., Bogdanov P.V., Churkin S.V., Dantchenko A.V., Devyatkin A.L., Murzin V.S., Samodurov G.D., Zhdanko A.B. (2000). Guide to the Butterflies of Russia and Adjacent Territories (Lepidoptera, Rhopalocera). Libytheidae, Danaidae, Nymphalidae, Riodinidae, Lycaenidae.

[30] Gaunet A., Dincă V., Dapporto L., Montagud S., Voda R., Schär S., Badiane A., Font E., Vila R. (2019). Two consecutive *Wolbachia*-mediated mitochondrial introgressions obscure taxonomy in Palearctic swallowtail butterflies (Lepidoptera, Papilionidae). Zool. Scr..

[31] Davlatov A.M., Lukhtanov V.A. (2025). *Pontia daplidice* (Linnaeus, 1758) and *Pontia edusa* (Fabricius, [1777]) in Tajikistan: One or two species? (Lepidoptera: Pieridae). SHIL. Rev. Lepidopterol..

[32] Gwiazdowska A., Rutkowski R., Sielezniew M. (2025). Conservation genetics of the endangered Danube Clouded Yellow butterfly *Colias myrmidone* (Esper, 1780) in the last Central European stronghold: Diversity, *Wolbachia* infection and Balkan connections. Insects.

[33] Kandul N.P., Lukhtanov V.A., Pierce N.E. (2007). Karyotypic diversity and speciation in *Agrodiaetus* butterflies. Evolution.

[34] Kocher T. (2004). Adaptive evolution and explosive speciation: The cichlid fish model. Nat. Rev. Genet..

[35] Santos M.E., Lopes J.F., Kratochwil C.F. (2023). East African cichlid fishes. EvoDevo.

[36] Stein F., Gailing O. (2025). Identification of BOLD engine deficiencies and suggestions for improvement based on a curated *Tachina* (Diptera) record set. PLoS ONE.

[37] Makhov I.A., Gorodilova Y.Y., Lukhtanov V.A. (2021). Sympatric occurrence of deeply diverged mitochondrial DNA lineages in Siberian geometrid moths (Lepidoptera, Geometridae): Cryptic speciation, mitochondrial introgression, secondary admixture or effect of *Wolbachia*?. Biol. J. Linn. Soc..

[38] Hinojosa J.C., Dapporto L., Brockmann E., Dincă V., Tikhonov V., Grishin N., Lukhtanov V.A., Vila R. (2021). Overlooked cryptic diversity in *Muschampia* (Lepidoptera: Hesperiidae) adds two species to the European butterfly fauna. Zool. J. Linn. Soc..

[39] Dubatolov V.V., Sergeev M.G., Zdanko A.B. (1994). New and little known species of the butterfly genus *Hyponephele* Muschamp, 1915. Atalanta.

[40] Eckweiler W., Bozano G.C. (2011). Guide to the Butterflies of the Palearctic Region: Satyrinae Part IV. Tribe Satyrini, Subtribe Maniolina: Maniola, Pyronia, Aphantopus, Hyponephele.

[41] Kramp K., Cizek O., Madeira P.M., Ramos A., Konvicka M., Castilho R., Schmitt T. (2016). Genetic implications of phylogeographical patterns in the conservation of the boreal wetland butterfly *Colias palaeno* (Pieridae). Biol. J. Linn. Soc..

[42] Pazhenkova E.A., Lukhtanov V.A. (2016). Chromosomal and mitochondrial diversity in *Melitaea didyma* complex (Lepidoptera, Nymphalidae): Eleven deeply diverged DNA barcode groups in one non-monophyletic species?. Comp. Cytogenet..

[43] Charlat S., Duplouy A., Hornett E.A., Dyson E.A., Davies N., Roderick G.K., Wedell N., Hurst G.D. (2009). The joint evolutionary histories of *Wolbachia* and mitochondria in *Hypolimnas bolina*. BMC Evol. Biol..

[44] Sucháčková B.A., Konvička M., Marešová J., Wiemers M., Ignatev N., Wahlberg N., Schmitt T., Fric Z.F. (2021). *Wolbachia* affects mitochondrial population structure in two systems of closely related Palaearctic blue butterflies. Sci. Rep..

[45] Ožana S., Dolný A., Pánek T. (2022). Nuclear copies of mitochondrial DNA as a potential problem for phylogenetic and population genetic studies of Odonata. Syst. Entomol..

[46] Tshikolovets V. (2011). Butterflies of Europe and the Mediterranean Area.

[47] Cheng Z., Li Q., Deng J., Liu Q., Huang X. (2023). The devil is in the details: Problems in DNA barcoding practices indicated by systematic evaluation of insect barcodes. Front. Ecol. Evol..

[48] Quaresma A., Ankenbrand M.J., Garcia C.A.Y., Rufino J., Honrado M., Amaral J., Brodschneider R., Brusbardis V., Gratzer K., Hatjina F. (2024). Semi-automated sequence curation for reliable reference datasets in ITS2 vascular plant DNA (meta-)barcoding. Sci. Data.

[49] Keller A., Hebert P. (2025). Automating the curation of DNA barcode databases for vascular plants. Environ. DNA.

[50] Kosterin O.E., Knyazev S.A. (2024). *Plusiodonta casta* (Butler, 1878) (Lepidoptera, Erebidae, Calpinae) found in West Sayan (Central Siberia, Russia). Acta Biol. Sibir..

