# Supplementary material for: Questionable Species Names for Distinct Species Clusters: An Empirical Test of the BOLD Molecular Identification Engine
Source: Insects. 2025 Nov 17;16(11):1172. doi: 10.3390/insects16111172 (PMC12653883; doi:10.3390/insects16111172)
Supplement: Supplementary file 1 [file insects-16-01172-s001.zip › Figure_S3.pdf]

BOLD TaxonID Tree

Title	:	Tree
Date	:	23-October-2025
Data Type	:	Nucleotide
Distance Model	:	Kimura 2 Parameter
Marker	:	COI-5P
Codon Positions	:	1st, 2nd, 3rd
Labels	:	Extra Info, Country & Province, Family
Filters	:	Length > 200
Attachment	:	Photographs & Spreadsheet

Sequence Count	:	26
Species count	:	2
Genus count	:	1
Family count	:	1
Unidentified	:	1

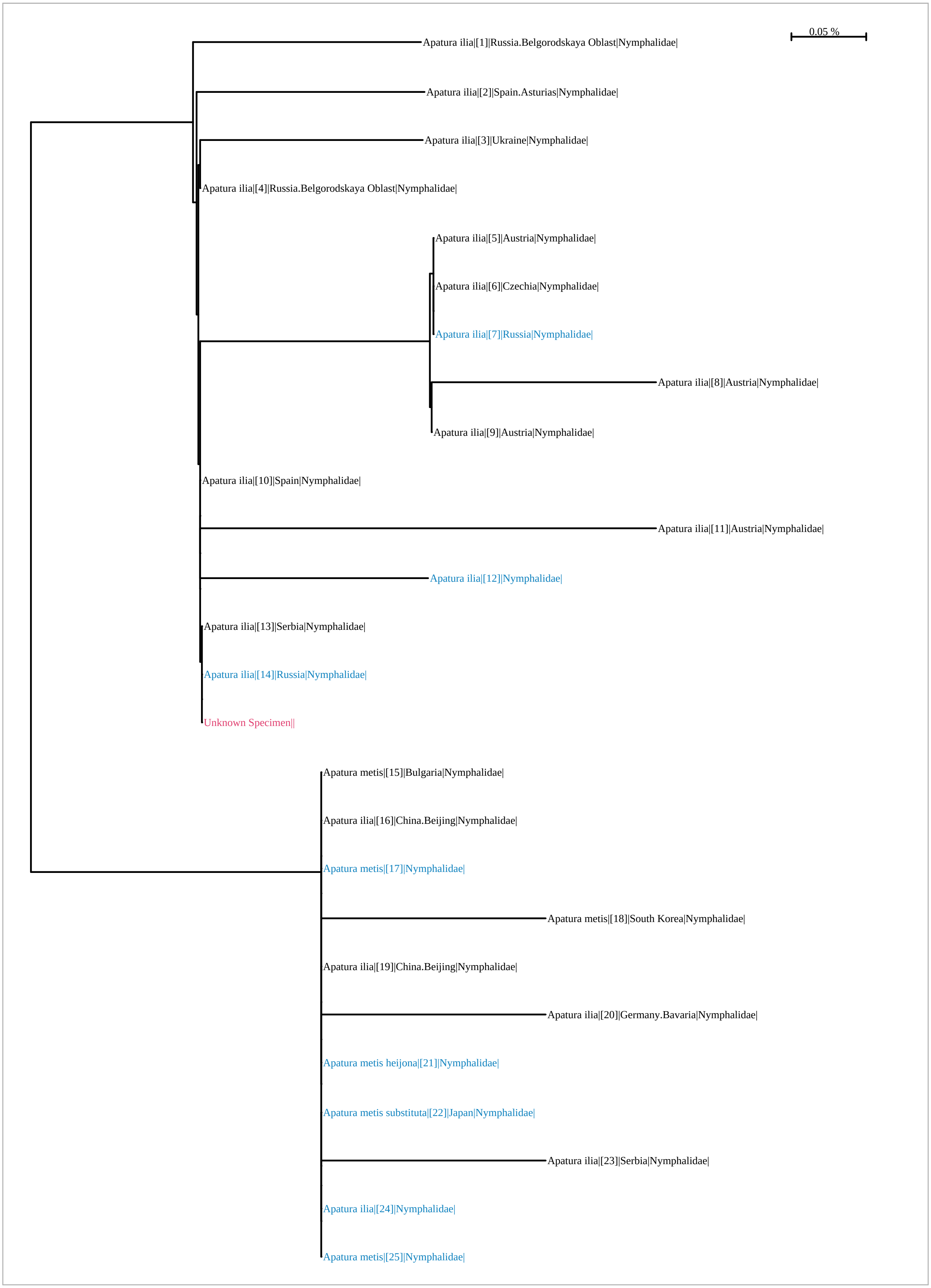
0.05 %
Apatura ilia|[1]|Russia.Belgorodskaya Oblast|Nymphalidae|
Apatura ilia|[2]|Spain.Asturias|Nymphalidae|
Apatura ilia|[3]|Ukraine|Nymphalidae|
Apatura ilia|[4]|Russia.Belgorodskaya Oblast|Nymphalidae|
Apatura ilia|[5]|Austria|Nymphalidae|
Apatura ilia|[6]|Czechia|Nymphalidae|
Apatura ilia|[7]|Russia|Nymphalidae|
Apatura ilia|[8]|Austria|Nymphalidae|
Apatura ilia|[9]|Austria|Nymphalidae|
Apatura ilia|[10]|Spain|Nymphalidae|
Apatura ilia|[11]|Austria|Nymphalidae|
Apatura ilia|[12]|Nymphalidae|
Apatura ilia|[13]|Serbia|Nymphalidae|
Apatura ilia|[14]|Russia|Nymphalidae|
Unknown Specimen||
Apatura metis|[15]|Bulgaria|Nymphalidae|
Apatura ilia|[16]|China.Beijing|Nymphalidae|
Apatura metis|[17]|Nymphalidae|
Apatura metis|[18]|South Korea|Nymphalidae|
Apatura ilia|[19]|China.Beijing|Nymphalidae|
Apatura ilia|[20]|Germany.Bavaria|Nymphalidae|
Apatura metis heijona|[21]|Nymphalidae|
Apatura metis substituta|[22]|Japan|Nymphalidae|
Apatura ilia|[23]|Serbia|Nymphalidae|
Apatura ilia|[24]|Nymphalidae|
Apatura metis|[25]|Nymphalidae|
