# Supplementary figures and images for: Questionable Species Names for Distinct Species Clusters: An Empirical Test of the BOLD Molecular Identification Engine
Source: Insects. 2025 Nov 17;16(11):1172. doi: 10.3390/insects16111172 (PMC12653883; doi:10.3390/insects16111172)

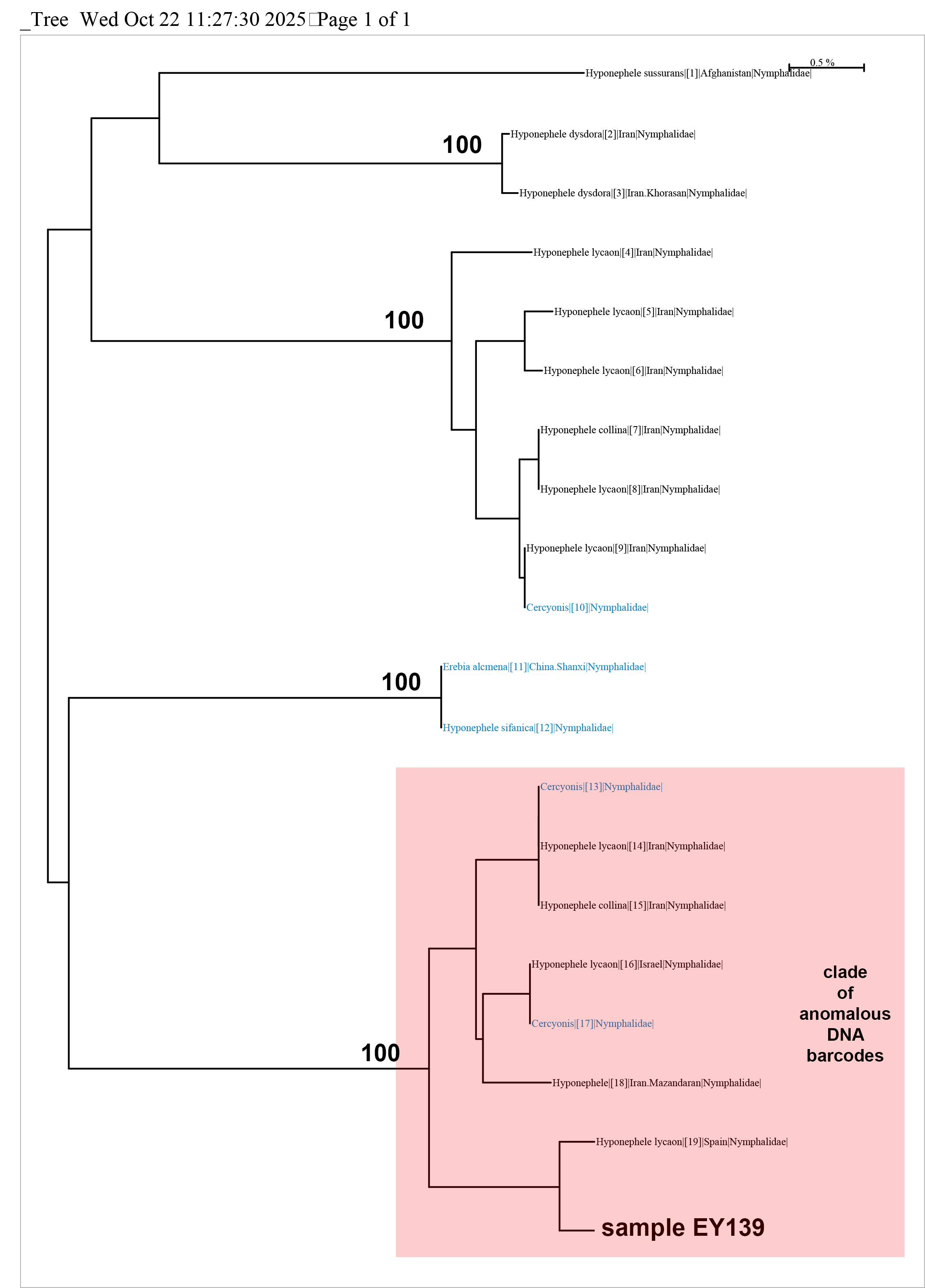

Supplement: Supplementary file 1 [file insects-16-01172-s001.zip › Figure_S1.tif]
